# Stereotactic body radiotherapy for bone oligometastatic disease in prostate cancer

**DOI:** 10.1007/s00345-019-02873-w

**Published:** 2019-07-25

**Authors:** Priyanka H. Patel, Cheng Lee Chaw, Alison C. Tree, Mansour Sharabiani, Nicholas J. van As

**Affiliations:** 1 Academic Unit of Radiotherapy and Oncology, Royal Marsden NHS Foundation Trust and Institute of Cancer Research, London, UK; 2 School of Public Health, Imperial College London, London, UK

**Keywords:** Prostate cancer, Stereotactic body radiotherapy, Oligometastatic disease, Bone metastases

## Abstract

**Purpose:**

There are sparse data describing outcomes of bone-only oligometastatic prostate cancer in comparison with lymph node disease treated with stereotactic body radiotherapy (SBRT). The primary aim of this study was to report progression-free survival (PFS) data for patients with bone-only disease. Influence of hormone sensitivity and androgen deprivation therapy use was also assessed.

**Methods:**

This is a single-centre retrospective cohort study. Hormone-sensitive and castrate-resistant patients with oligometastatic (≤ 3) bone-only prostate cancer treated with SBRT were included. Data were collected using electronic records. Kaplan–Meier survivor function, log rank test, as well as Cox regression were used to calculate PFS and overall survival.

**Results:**

In total, 51 patients with 64 bone metastases treated with SBRT were included. Nine patients were castrate resistant and 42 patient’s hormone sensitive at the time of SBRT. Median follow-up was 23 months. Median PFS was 24 months in hormone-sensitive patients and 3 months in castrate-resistant patients. No patients experienced grade 3 or 4 toxicities. There were three in-field recurrences.

**Conclusions:**

In this study, patients with bone oligometastatic disease showed potential benefit from SBRT with a median PFS of 11 months. Hormone-sensitive patients showed the greatest benefit, with results similar to that published for oligometastatic pelvic nodal disease treated with SBRT. Prospective randomised control trials are needed to determine the survival benefit of SBRT in oligometastatic bone-only prostate cancer and to determine prognostic indicators.

**Electronic supplementary material:**

The online version of this article (10.1007/s00345-019-02873-w) contains supplementary material, which is available to authorized users.

## Background

Oligometastatic disease (OMD) was first described in 1995 by Hellman and Weischselbaum as a transitional state between a solitary localised tumour and widespread metastatic disease [1]. The clinical significance of recognising OMD is the ability to ablate or surgically remove these lesions with the potential of improving survival and delaying further metastases, or even curing the patient.

Understanding of OMD has evolved greatly, since it was first described. Oligometastases are identified more, as patients are being imaged frequently with advanced modalities. There is no unified consensus on the maximum number of metastases which defines OMD. Oligometastatic prostate cancer has generally been classified as ≤ 3 metastases in bone or lymph nodes only, whilst other criteria have used up to six [2].

Treatment options for polymetastatic disease have recently been updated. Immediate or delayed androgen deprivation therapy (ADT) has been the established first-line treatment strategy with the introduction of chemotherapy or ADT such as abiraterone or enzalutamide once patients become castrate [3, 4]. More recently, data have shown a further survival benefit with upfront docetaxel or abiraterone in hormone-sensitive patients [5, 6].

Management of oligometastatic prostate cancer is a current area of great interest. Prostate cancer commonly metastasises to bone, and up to 63.6% of patients have bone-only metastases [7]. The optimal management strategy in this patient group is uncertain owing to a lack of prospective randomised control trials comparing standard of care with ablative therapies. In the UK, through a prospective service evaluation programme, patients are being managed with SBRT for oligometastases with ≤ 3 sites of disease [8]. Patients with any site of OMD can be treated with SBRT, but the data are sparse on the behaviour of bone OMD and suitability for radical treatment.

The optimal use of ADT in oligometastatic prostate cancer is not clear. However, SBRT is an effective ablative delivery of radiotherapy with minimal toxicity, achieving 80–90% local control rates when using biological effective doses (BED) of > 100 Gy [9, 10].

Despite a paradigm shift in the management of polymetastatic prostate cancer, the treatment of patients with oligometastases remains varied between institutions. STOMP, a multicentre phase 2 trial has reported an increased ADT-free survival in patients treated with SBRT for oligometastases [11]. The results of this trial have laid the foundations for a phase 3 randomised control trial. Further supporting data are needed to determine whether bone oligometastatic prostate cancer should be treated with the same approach as lymph node disease.

The purpose of this study was to determine the outcomes and tolerability of SBRT in bone-only OMD in patients with prostate cancer. The primary aim was to evaluate progression-free survival (PFS). Secondary aims included acute toxicity, overall survival (OS), local progression-free survival (LPFS), and influence of ADT use with SBRT.

## Methods

Patients with metastatic bone-only prostate cancer with ≤ 3 sites of disease, presenting with metachronous or synchronous disease to the Royal Marsden Hospital between July 2011 and March 2018 were identified from a retrospective cohort analysis using hospital electronic patient records, and 11 of these patients have been previously included in the study by Ost et al. [12].

Patients were included if they were hormone naïve, hormone sensitive, or castrate resistant at the time of SBRT. Patients could have received concomitant ADT ± chemotherapy with SBRT. Patients were excluded if their primary disease was not treated radically. Data including patient characteristics, symptoms, treatment doses, and outcomes were collected.

Image fusion, immobilisation, dose and fractionation, and tumour tracking methods were determined following discussion within an SBRT multi-disciplinary meeting. Tracking methods included CT-guided insertion of one to three gold seeds into adjacent soft tissue for tracking rotational and translational movements. Dose constraints were determined using organ at risk constraints from UK SABR consortium guidelines [13].

All patients were treated with SBRT using Cyberknife radiotherapy; 2 (3.9%) patients were treated using a C-arm linac machine as well for a separate metastasis.

Most patients had diagnostic imaging fused to assist accurate delineation. Twenty-five (39.1%) bone metastases were delineated with MRI fusion. Nineteen (29.7%) metastases were planned using PET/CT fusion, 18 (28.1%) metastases had both PET and MRI fused, and 3 (4.7%) patients had 4DCTs for planning.

All treatments were completed over 3–11 days. Prescription doses were determined by meeting organs at risk dose constraints. Treatment details are tabulated in Table 1.

**Table 1:** Description of treatment site and technique

	Number of bone metastases treated = 64 Number of patients = 51
Hormone-sensitive	42 (82.4%)
Castrate-resistant	9 (17.6%)
Metachronous	54 (84.4%)
Synchronous	10 (15.6%)
*Metastatic site*	
Sternum	1 (1.6%)
Cranium	1 (1.6%)
Scapula	3 (4.7%)
Humerus	2 (3.1%)
Ribs	5 (7.8%)
Spine	28 (43.8%)
Pelvis	23 (35.9%)
Femur	1 (1.6%)
*Number of metastases per patient* (%)	
1	40 (78.4%)
2	9 (17.3%)
3	2 (3.8%)
*Number of bone metastases identified by imaging modality* (%)	
Choline PET/CT	41 (64%)
WBDWIMRI	6 (9.4%)
PET/CT and WBDWMRI	5 (7.8%)
NM bone scan and SPECT or CT	2 (3.1%)
PSMA PET/CT	7 (10.9%)
MRI and CT	1 (1.6%)
CT	1 (1.6%)
WBDWIMRI and CT	1 (1.6%)
*Tumour tracking* (%)	
Fiducials	19 (29.7%)
X Sight spine	42 (65.6%)
6D Skull	1 (1.6%)
CBCT	2 (3.1%)
*Doses/fractionation* (%)	
24 Gy/3 fractions	4 (6.3%)
27 Gy/3 fractions	4 (6.3%)
30 Gy in 5 fractions	3 (4.7%)
30 Gy in 3 fractions	53 (82.8%)

*PET/CT* positron-emission tomography–computed tomography, *WBDWIMRI* whole-body diffusion-weighted magnetic resonance imaging, *NM* nuclear medicine, *SPECT* single-photon-emission computed tomography, *CBCT* cone-beam-computed tomography, *PSMA* prostate-specific membrane antigen

Progression-free survival and overall survival were calculated using Kaplan–Meier method and log rank test. Progression-free survival was defined as time between SBRT and the first of the following to occur; biochemical PSA, radiological or symptomatic progression or death. Biochemical failure was defined as PSA increase ≥ 25% and > 2 ng/ml above nadir or, in case of no decline, then ≥ 25% and ≥ 2 ng/ml increase above baseline at least 12 weeks after treatment. Radiological progression was defined as a new lesion with standardised uptake value (SUV) above normal physiological background found on PET/CT imaging using choline or prostate-specific membrane antigen radiotracers, or new lesion identified on whole-body diffusion-weighted MRI (WBDWMRI).

Univariate and stepwise multivariable Cox regression analyses were performed using Stata 15.1 for Windows.

Overall survival was measured from SBRT to last follow-up or death. Local progression-free survival was defined from SBRT to date of local progression on PET/CT with increased size or SUV ≥ 12 weeks after SBRT completion. Acute toxicity was defined as symptoms presenting within 3 months.

## Results

In total, 51 patients with 64 bone metastases treated with SBRT were included. Nine patients with 11 bone metastases were castrate-resistant at diagnosis, and the remaining 42 patients were hormone-sensitive at diagnosis of OMD. Forty-three patients presented with metachronous OMD and eight with synchronous OMD.

One patient had a bone metastasis at the sacro-iliac joint included in the primary prostate radiotherapy field. The majority of patients (40 patients, 78.4%) were diagnosed with advanced stage disease (stage III/IV) at presentation. For metachronous disease, the median (IQR) time from completion of primary treatment to presentation with oligometastases was 40.5 (14–68) months. One patient presented with two consecutive oligometastases 9 months apart. The baseline characteristics of the cohort are tabulated (Table 2).

**Table 2:** Patient characteristics at initial diagnosis

	Number of patients = 51
Median age	67.5 (43–83)
*PSA at initial presentation* (%)	
Range	3.8–360
<20	32 (62.7%)
>20	16 (31.3%)
Unknown	3 (5.8%)
Metachronous	43 (84.3%)
Synchronous	8 (15.7%)
*Gleason grade grouping* (%)	
No biopsy available	5 (9.8%)
1	4 (7.8%)
2	4 (7.8%)
3	14 (27.5%)
4	6 (11.8%)
5	18 (35.3%)
*ADT treatment at primary diagnosis*	
Yes	39 (76.5%)
No	12 (23.5%)
*Primary tumour staging*	
Unknown	2 (3.9%)
T1	0
T2	10 (19.6%)
T3	38 (74.5%)
T4	1 (1.9%)
*Nodal staging*	
Unknown	2 (3.9%)
N0	39 (76.5%)
N1	10 (19.6%)
*Metastatic disease*	
M0	42 (82.4%)
M1	9 (17.6%)
*Primary treatment*	
Radical prostatectomy alone	5 (9.8%)
Radical radiotherapy to prostate ± pelvic lymph nodes	23 (41.2%)
Radical prostatectomy and prostate bed ± pelvic lymph node radiotherapy	23 (39.2%)
Salvage cystoprostatectomy/pelvic exenteration	5 (9.8%)
Salvage HIFU	1 (1.9%)

*HIFU* high intensity focused ultrasound

Median (IQR) time from diagnosis of oligometastasis to starting radiotherapy was 3 (1–6) months.

Median (IQR) PSA prior to starting any treatment after diagnosis of OMD was 5.3 (7.3–35) ng/ml.

### Progression-free survival

Median (IQR) follow-up from last radiotherapy treatment was 23 (10–32.3) months. At the time of analysis, median PFS was 11 (95% CI 8–25) months. At 1 year, 23/51 (45.1%) patients had progressed; at 2 years, a further two of the remaining 28 (7.1%) patients had progressed.

**Fig. 1:**
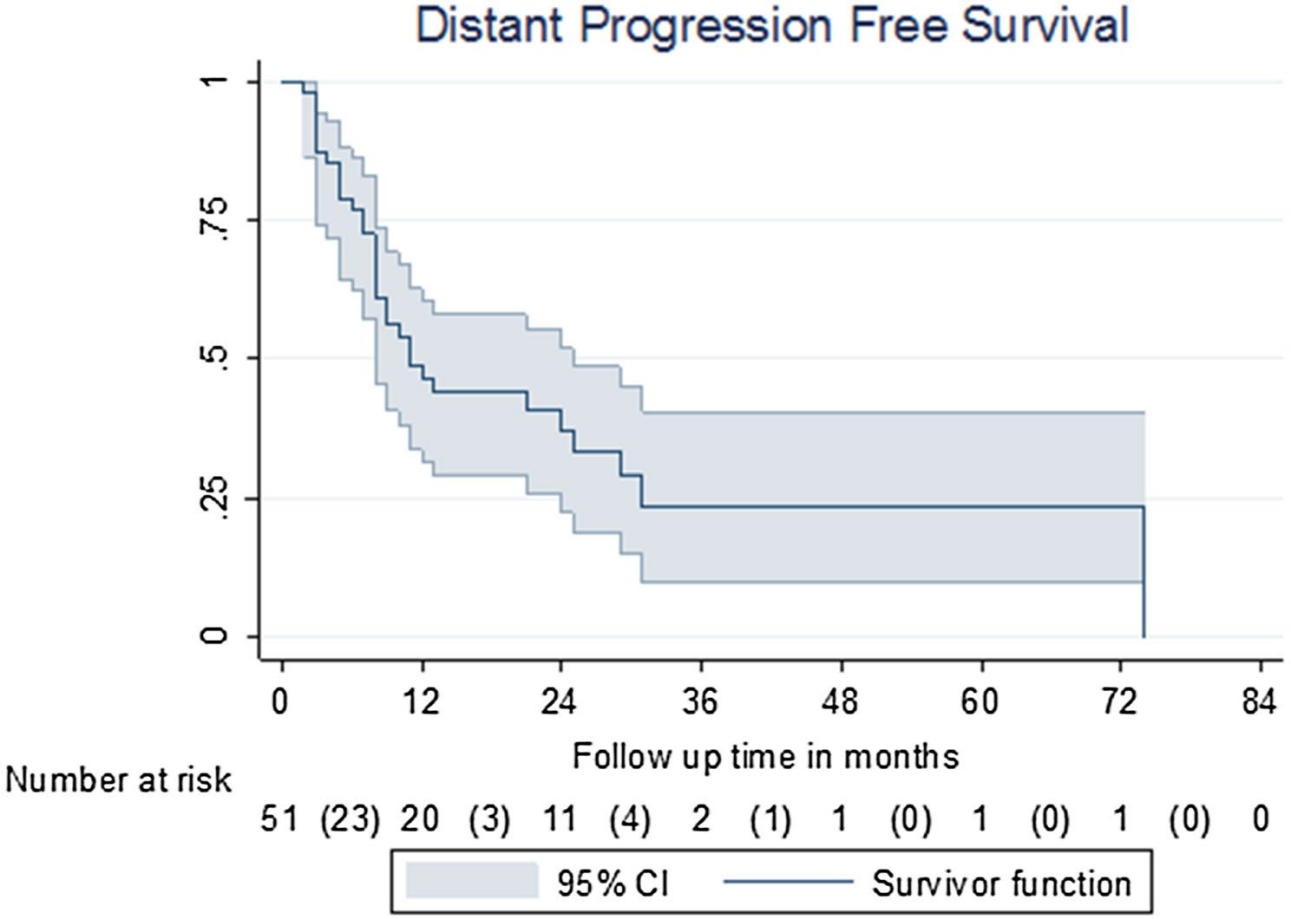
Disease progression-free survival

**Fig. 2:**
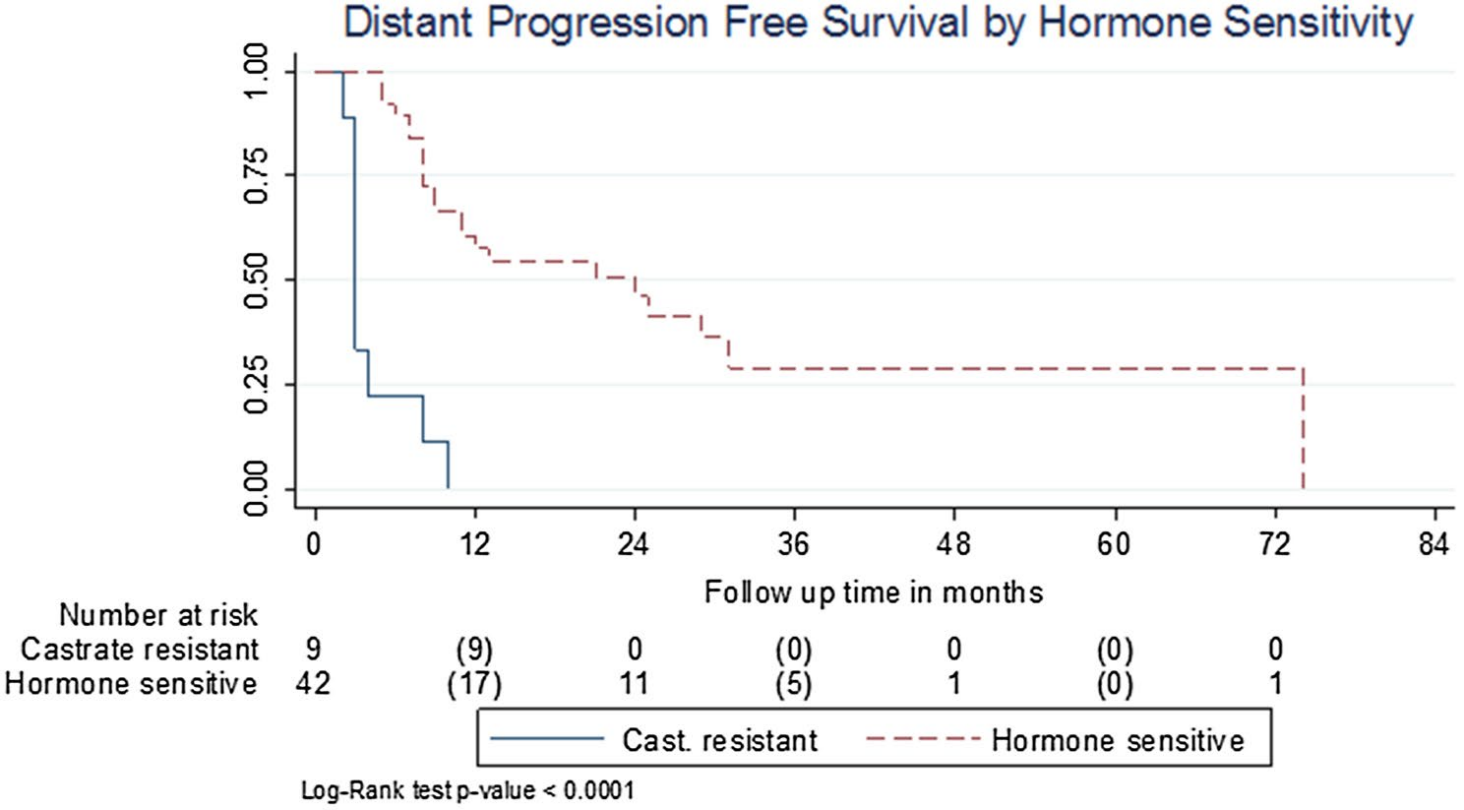
PFS for castrate- vs hormone-sensitive patients

Progression-free survival was significantly different between patients with castrate-resistant and hormone-sensitive cancer at the time of SBRT, with median PFS being 3 (95% CI 2–8) months and 24 (95% CI 9–31) months, respectively (*P* < 0.0001) (Figs. 1, 2).

Of the patients who remained hormone-sensitive at diagnosis of oligometastases, 33 patients (42 metastases) were treated with SBRT + ADT, and 9 patients (11 metastases) had no ADT. Median concomitant ADT length at the time of analysis was 6 (1–52) months. Sixteen patients completed a short-course (< 6 months) of ADT, median 4 (2–6) months, 17 patients received long-course ADT (> 6 months), and median 23 (7–52) months. There was a significant difference in PFS between no ADT, short-term ADT and long-term ADT after SBRT, on log rank test (*P* = 0.015). (Supplementary material Fig. 1).

Univariate Cox regression analysis indicated a significant association between PFS and long-term ADT HR 0.24 (95% CI 0.08–0.71) and a very weak association between PFS and short-term ADT HR 0.37 (95% CI 0.13–1.09). No association was found on multivariable analysis. Both univariate and multivariable Cox regression analyses indicated a significant association between PFS and castrate resistance, HR 8.43 (95% CI 3.47–20.45, *P* < 0.001). No significant association between age, presenting PSA, PSA prior to SBRT or metachronous disease was found.

### Overall survival

During the follow-up period, four patients (7.8%) died. All four were castrate-resistant at the time of SBRT treatment. Median OS was not yet reached. One, 2-, 3-, and 4-year OS rates were 97%, 97%, 92.6%, and 73%, respectively (Supplementary material Fig. 2).

### Local recurrence

Three (5.9%) patients relapsed in field; these patients relapsed at 4, 23, and 30 months from treatment, respectively. Sites of relapse included the scapula, rib, and thoracic vertebra. Median LPFS was not yet reached. 1-, 2-, and 3-year local-free recurrence rates were 98%, 95%, and 90%, respectively (Supplementary material Fig. 3).

### Symptomatic outcomes

48 patients completed 6 month follow-up, and no patients experienced grade 3 or 4 toxicities. One patient had reduced pain from grade 2 to grade 1, and two patients had grade 1 pain at baseline which resolved with radiotherapy. Three patients continued to have grade 1 pain which started after SBRT. One patient developed grade 2 pain due to a fracture at the treated site, which resolved, but was exacerbated at 6 months by a proceeding fall. There was one long-term toxicity of pain radiating down the leg from treatment to L5 lesion (Supplementary material Table 1).

## Discussion

This study has found that SBRT is a well-tolerated therapy in patients with oligometastatic bone prostate cancer. The median PFS was 24 months in hormone-sensitive patients and this is similar to the current evidence base. Results are also comparable to available published retrospective data on treating lymph node disease with SBRT [14–16], therefore, suggesting that OMD of bone in prostate cancer patients may benefit from SBRT treatment. This is a promising management strategy warranting further investigation.

The standard management of oligometastatic prostate cancer in the UK is systemic treatment with ADT with docetaxel chemotherapy. Androgen deprivation therapy is associated with increased morbidity and side effects impacting quality of life [17]. Intermittent ADT reduces side effects and is considered to be non-inferior to continuous ADT [18, 19]. Therefore, methods to delay ADT use are needed to defer significant side effects as well as to improve PFS. Patients with fewer metastases (≤ 3) have been shown to have a longer OS compared to patients with polymetastatic disease [20, 21]; these patients potentially represent a different biology of disease which may benefit from SBRT to delay further progression and influence OS.

Long-term follow-up of the RADAR phase 3 trial [22] has shown that bone OMD is common. Of the 176 patients identified with metastatic bone disease, and 55 patients had ≤ 3 bone metastases.

There have been several retrospective studies which have reviewed the use of SBRT in oligometastatic prostate cancer and have shown positive results [16, 23, 24]. Ost et al. reported a median PFS of 21 months (95% CI 15–27 months) with 70% of patients having ≤ 3 metastases at progression [12]. There was no statistically significant difference between ADT and no ADT with SBRT with median PFS of 25 vs 18 months. Patients included both bone and pelvic lymph node OMD. A multicentre study found median PFS in oligometastatic prostate cancer treated with SBRT to be 17.7 months; it was lower in patients with bone metastases compared to lymph nodes although not statistically significant (11 months vs 21.4 months) [16]. A retrospective study has investigated the effectiveness of Cyberknife in patients with prostate cancer oligometastatic bone-only disease [25]. The study included 51 patients with 71 bone metastases and found OS at 1, 2, and 3 years was 90%, 76%, and 70%, respectively. These studies support the use of SBRT in prostate bone OMD along with the results found within this study. Further work will clarify prognostic indicators to identify those patients with bone OMD most likely to benefit from SBRT.

## Limitations

We acknowledge that there are a number of limitations to our study that limit the strength of the conclusions that can be drawn. This was a retrospective study using routinely captured data from electronic patient records. There is considerable heterogeneity amongst patient characteristics and treatment pathways. The study includes a small number of patients which may limit the strength of the conclusions drawn. The majority of patients had PET/CT scans which have been shown to have a high specificity in detecting prostate cancer metastases [26]; however, biopsy confirmation was not performed for histological confirmation, in line with current standard practice.

## Conclusion

Stereotactic body radiotherapy is a well-tolerated treatment with minimal and tolerable side effects in OMD [9]. The majority of studies show SBRT to be well tolerated amongst elderly metastatic prostate cancer patients [16, 22, 25].

This study shows similar outcomes to those presented in current literature of groups of patients with heterogeneous sites of metastases. Our data suggest that men with bone-only oligometastatic disease have good outcomes after SBRT to all sites of disease. On-going prospective randomised studies will clarify the magnitude of benefit from SBRT.

## Electronic supplementary material

Below is the link to the electronic supplementary material.

Supplementary material 1 (DOCX 1644 kb)
