# Supplementary material for: Stereotactic body radiotherapy for bone oligometastatic disease in prostate cancer
Source: World J Urol. 2019 Jul 25;37(12):2615–21. doi: 10.1007/s00345-019-02873-w (PMC6868044; doi:10.1007/s00345-019-02873-w)
Supplement: Supplementary file 1 — Supplementary material 1 (DOCX 1644 kb) [file 345_2019_2873_MOESM1_ESM.docx]

Supplementary material

Figure 1: PFS in patients treated with SBRT+ short-term ADT vs ADT + long-term ADT vs SBRT only (excluding castrate resistant patients).


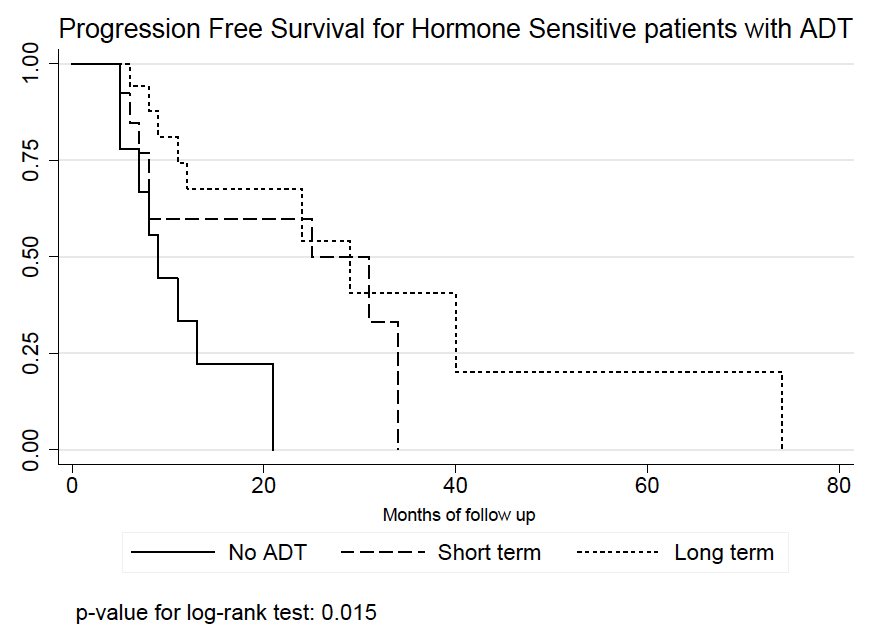


Figure 2: Overall Survival
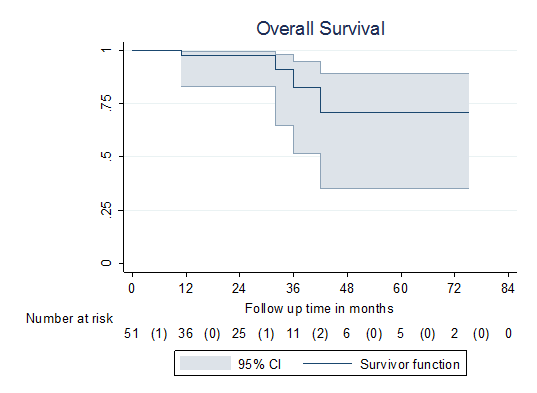


Figure 3: Local Progression free survival


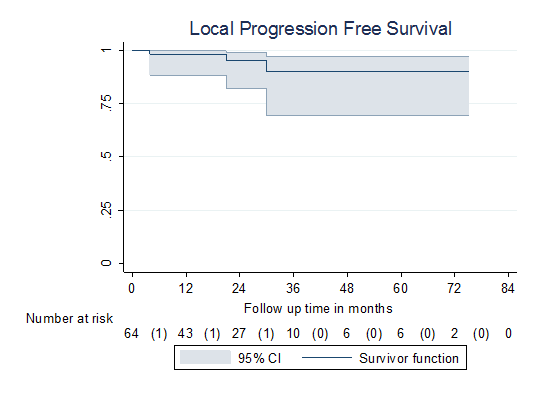


Table 1: Toxicity assessment at 6 weeks, 3 and 6 months post treatment, side effects graded as per CTCAE 4.0

| Toxicity | 4-6 weeks |  | 3months |  | 6 months |  |
| --- | --- | --- | --- | --- | --- | --- |
|  | Grade 1 | Grade 2 | Grade 1 | Grade 2 | Grade 1 | Grade 2 |
| Fatigue | 13 | 3 | 1 |  | 1 |  |
| Cough | 1 | 0 |  |  |  |  |
| Bowel disturbance/proctitis | 3 | 0 |  |  |  |  |
| Pain | 5 | 1 | 5 | 1 | 4 |  |
| anorexia | 1 | 0 |  |  |  |  |
| Oesophagitis | 1 | 0 |  |  |  |  |
| Nausea | 1 | 0 |  |  |  |  |
| Dysphagia | 1 | 0 |  |  |  |  |
| Haematuria | 1 | 0 |  |  |  |  |
| Fracture | 0 | 1 |  |  |  |  |
